# Model of the Long-Term Transport and Accumulation of Radionuclides in Future Landscapes

**DOI:** 10.1007/s13280-013-0402-x

**Published:** 2013-04-26

**Authors:** Rodolfo Avila, Ulrik Kautsky, Per-Anders Ekström, Per-Gustav Åstrand, Peter Saetre

**Affiliations:** 1 Facilia AB, Gustavslundsvägen 151G, 167 51 Bromma, Sweden; 2 Swedish Nuclear Fuel and Waste Management Co. (SKB), Box 250, 101 24 Stockholm, Sweden

**Keywords:** Radionuclides, Model, Biosphere, Radiation dose, Radioactive waste, Repository

## Abstract

Assessments of radiological impacts on humans and other biota from potential releases to the biosphere from a deep geologic repository for spent nuclear fuel are associated with several challenges. Releases, if any, will likely occur in a far future and to an environment that will have experienced substantial transformations. Such releases would occur over very long periods during which environmental conditions will vary continuously due to climate change and ecosystem succession. Assessments of radiological impacts must therefore be based on simulations using models that can describe the transport and accumulation of radionuclides for a large variety of environmental conditions. In this paper we describe such a model and show examples of its application in a safety assessment, taking into account results from sensitivity and uncertainty analyses of the model predictions.

**Electronic supplementary material:**

The online version of this article (doi:10.1007/s13280-013-0402-x) contains supplementary material, which is available to authorized users.

## Introduction

A major task in the safety assessment of a deep geological repository for nuclear waste is to demonstrate that it remains safe for humans and the environment for a time period in the range of several hundred thousand to one million years into the future. Models used for such assessments should take into account that potential releases to ecosystems may occur in the far future, by which time the ecosystems will have undergone considerable changes. The aim of this article is to describe the model of the long-term transport and accumulation of radionuclides in the biosphere, hereafter called the Radionuclide Model of the biosphere, that was used for dose estimations in ‘SR-Site’ (SKB 2011), a safety assessment undertaken by the Swedish Nuclear Fuel and Waste Management Company (SKB) of a future geological repository for spent nuclear fuel in Sweden.

Given the requirements of the safety assessment and the features of the potentially impacted environments, the Radionuclide Model of the biosphere should be capable of: (i) addressing temporal changes in ecosystems driven by climate change, land rise, shoreline displacement, and ecosystems succession, (ii) handling time-dependent releases with groundwater discharges that are heterogeneously distributed across a landscape, (iii) handling releases of many radionuclides with different geochemical behavior and subject to radioactive decay, some giving rise to daughter radionuclides, (iv) assessing exposures of humans and wildlife to radionuclides heterogeneously distributed in the landscape, and (v) taking into account transport of radionuclides between different areas of a potentially impacted landscape driven by surface and subsurface runoff.

In a previous safety assessment (SKB 2006) we developed a model of the fate of radionuclides in the whole potentially affected landscape, which takes into consideration temporal transformations of that landscape and radionuclide transport processes within it (Avila et al. 2006). The Radionuclide Model of the biosphere briefly described in this article is a further development of the Avila et al. (2006) model and incorporates new results from site investigations and new developments in climate models (Näslund et al. 2013), hydrological models (Berglund et al. 2013a), and landscape models (Lindborg et al. 2013) that have been obtained within the SR-Site safety assessment (see overview in Kautsky et al. 2013). For a full description of the Radionuclide Model of the biosphere, see Avila et al. (2010). Here we also present examples of applications of the model for assessments of doses to humans. Results of dose assessments for other biota than humans are provided by Torudd and Saetre (2013). There are also alternative models for the marine ecosystems (Erichsen et al. 2013).

## Materials and Methods

### Approach to Model Development

The main principles used for developing this model are generally the same that we applied in the development of the model described in Avila et al. (2006). Essentially, we consider that for a model to have all required capabilities mentioned above, it should describe the processes of transport and accumulation in the biosphere at a landscape level, rather than for each ‘biosphere object’ separately. The reasons for this are discussed in Avila et al. (2006).

A ‘biosphere object’ is defined as an area of the landscape that can receive radionuclide releases; either through discharge of deep groundwater (Berglund et al. 2013a) or in contaminated surface water, at any time during a glacial cycle (Lindborg et al. 2013). The identification of biosphere objects is described by Lindborg et al. (2013).

In our earlier model, we divided the potentially affected landscape into several interlinked biosphere objects. For each of these objects we used several radioecological models, since we expected that at different future times different ecosystem types will prevail in a biosphere object and a single radioecological model that could handle this was not available. By radioecological model we mean a model that can simulate the transfer and accumulation of radionuclides in an ecosystem. In that previous model we handled transitions between ecosystems by introducing discrete events, i.e., at a given time we substituted one radioecological model with another one during the simulations. We then transferred the accumulated inventory between the ecosystem models.

This created several problems, mainly because the different radioecological models had different compartments, which made it difficult to ensure mass balance at the transitions. Also, the transition between ecosystems is a continuous process and treating it as a discrete event creates unrealistic abrupt changes in predicted time series of inventories and concentrations, which are difficult to handle numerically. Another problem with the previous model was that we used existing radioecological models, although with some modifications (Avila 2006a, b) and some of these models did not include all relevant processes. In particular, processes related to the transport and accumulation of radionuclides in the lower regolith, e.g., till from the last glaciations, were not included.

The model that we describe in the “Results and Discussion” section is an attempt to solve the above-mentioned problems, by modeling each biosphere object with a single radioecological model that is applicable to all those ecosystem types that are of relevance for the given assessment context. We also impose the requirement that the model should be able to simulate transitions between ecosystems in a continuous manner, rather than as discrete events. As we show later in this paper, a main idea in this model is that it includes an aquatic and a terrestrial part that interact with each other, through fluxes of water and particles and processes related to biomass production.

**Fig. 1:**
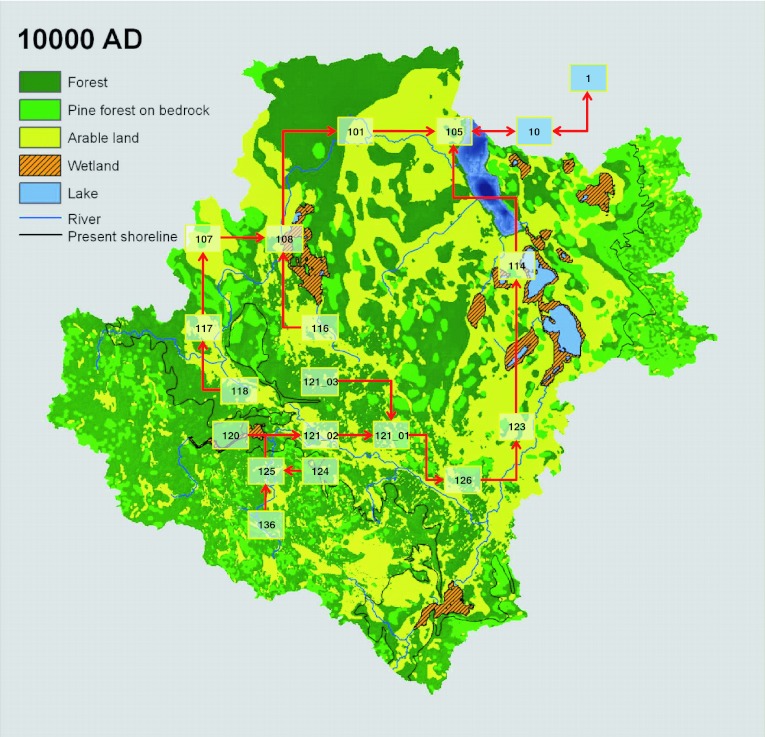
Illustration of the landscape model at 10 000 ad (from Lindborg 2010). The *boxes* show biosphere objects (with *identity numbers*) at their approximate locations in the landscape and *red arrows* indicate the surface water flow paths connecting the objects. The *blue boxes* represent the combined objects of Öregrundsgrepen (object 10) and the model area outlet, the Baltic Sea (object 1)

Figure 1 shows an example of the landscape model at a specific point in time within a glacial cycle (Näslund et al. 2013). The boxes show the different biosphere objects and the arrows the fluxes of water and particles, as well as of radionuclides, between them. Since the biosphere objects are interconnected, the model can simulate the transport and accumulation of radionuclides in the whole landscape. In the terrestrial phase, when biosphere objects have emerged from the sea and have been converted to lakes and wetlands, the radionuclide fluxes from a biosphere object are directed to connected downstream objects. Hence, all downstream objects will receive inputs from one or several upstream objects. In the Sea Stage, when biosphere objects are still sea basins, these interact only with the outer coastal area (Öregrundsgrepen) via water exchange in both directions. From Öregrundsgrepen, radionuclides are finally discharged to the Baltic Sea.

### Estimation of Model Parameter Values

Extensive site investigations at the site selected for a repository for spent nuclear fuel in Sweden, Forsmark, have resulted in a detailed description of the site and its development (SKB 2011). Data obtained from the site investigations together with a better understanding of the site has been the primary source for parameter values of the Radionuclide Model of the biosphere. Parameters represent the development of the individual biosphere objects, characteristics of the relevant ecosystems, and water flows within and between biosphere objects. There are also parameters describing the exposed individuals and dose coefficients for external exposure, inhalation, and ingestion of food and water. For each parameter, we derived a best estimate (BE) value using site and/or literature data, and to describe the uncertainty of parameter values we assigned a probability density function (PDF) to those parameters that are considered uncertain; i.e., to most parameters excluding those that are considered constants, such as the dose coefficients that relate radiation dose to intake of activity. In the safety assessment, we used the BE values in deterministic simulations to obtain baseline dose estimates (see below) and the PDFs in probabilistic simulations to assess the impact of parameter uncertainty on the dose estimates.

For most parameters, we selected BE values and PDFs using expert judgment as described by Avila et al. (2010). Two element-specific parameters, the distribution coefficients (*K*_d_) and concentration ratios (CR), are known from previous safety assessments (SKB 2006) to have a large impact on the dose predictions. The *K*_d_ and CR are used to model radionuclide retention and biological uptake, respectively. For these parameters, we applied a more formalized approach for derivation of BE values and PDFs using Bayesian inference methods (Nordén et al. 2010). These methods allow combination of site and literature data in a systematic way and with a good theoretical basis. The site investigations included extensive measurements of *K*_d_ and CR values (Nordén et al. 2010) for a large number of elements, including those corresponding to the most important radionuclides, like ^226^Ra and ^129^I. We retrieved literature data primarily through the EMRAS (IAEA 2010) and ERICA databases (Beresford et al. 2007). Where relevant values were missing in these databases, we used values compiled for previous SKB safety assessments (Karlsson and Bergström 2002). Finally, where appropriate data were not available from the site or from the open literature we used data for analog biota types or elements.

### Simulations for Dose Assessments

The model accepts time and spatially distributed releases as input. However, for the purpose of this specific safety assessment we performed simulations for unit releases of each radionuclide to obtain radionuclide-specific Dose Conversion Factors (annual doses per unit release rate), which we then multiplied by the actual releases of the corresponding radionuclide to obtain radiation dose values resulting from those releases. The Swedish regulations (SSM 2008a, b) require that annual dose averaged over the lifetime of the individuals be calculated for comparison with the risk criteria. This means that it is not necessary to calculate doses to different age groups, as this average can be adequately represented by the annual dose to an adult (ICRP 2000). Hence, in the derivation of Dose Conversion Factor values, we have calculated doses to adults averaged over a lifetime. The term dose is taken to mean ‘effective dose’, including, as appropriate, the committed dose from intakes of radionuclides and the contribution from external irradiation.

For this study we derived two different Dose Conversion Factors: (i) the Landscape Dose Conversion Factor (LDF) that is applicable to continuous long-term releases over hundreds to tens of thousands of years at a constant rate and is defined as the annual effective dose to a representative individual from the most exposed group resulting from a constant unit release rate of this radionuclide to the biosphere (the units are Sv a^−1^ per Bq a^−1^) and (ii) the Landscape Dose Conversion Factor for pulse releases (LDF pulse) that is applicable to a radionuclide release that reaches the biosphere in a pulse over years to hundreds of years and is defined as the annual effective dose to a representative individual from the most exposed group resulting from a unit pulse release of this radionuclide to the biosphere (the units are Sv a^−1^ per Bq). The most exposed group is defined as the group of individuals exposed to the biosphere object with the potentially highest contamination, considering a glacial cycle from a submerged landscape to fully terrestrial conditions. A representative individual from the most exposed group is assumed to spend all time in this biosphere object, and get his/her entire supply of food and water from the object, but still considering constraints in water and food supply (Kautsky et al. 2013; Saetre et al. 2013). All potential exposure pathways are considered in the dose calculations, including external irradiation from radionuclides in the surrounding environment and internal irradiation from radionuclides incorporated into the human body by inhalation and via ingestion of food and water.

We calculated LDF values for three different periods of the reference glacial cycle (Näslund et al. 2013): the period of submerged conditions following the deglaciation, the interglacial period, and a prolonged period of periglacial conditions. Additionally, we calculated LDFs for a global warming climate case. The maximum LDF in the landscape during each time period was used in the dose calculations for the safety assessment.

The first part of the reference glacial cycle is represented by temperate conditions, i.e., climate conditions similar to those of today. This interglacial period is assumed to exist for 18 400 years (i.e., at the first occurrence from −9000 ad to 9400 ad, but then recurring in each glacial–interglacial cycle). When the period starts, the landscape is covered by the sea (submerged conditions). As land emerges sufficiently out of the sea, wetlands are first created and then possibly converted to arable land (Lindborg et al. 2013). The interglacial period with temperate conditions is followed by a period with periglacial conditions, representing a colder climate than today with deep permafrost. These conditions are assumed to prevail for 50 200 years (i.e., from 9400 ad to 59 600 ad at the first occurrence). During glacial conditions, when the repository is covered by an inland ice sheet, releases can only cause humans to be exposed to radionuclides through ingestion of sea food, when the ice margin is situated above or close to the repository. The resulting doses in this case are expected to be lower than maximum doses during temperate conditions, due to a larger dilution of radionuclides released to the sea. To estimate annual exposures from releases during glacial conditions, the LDFs from the open-sea stage (submerged period) obtained for temperate conditions are used in the assessment.

For the global warming climate case we assumed that global warming will extend the period of temperate conditions, which will prevail during the whole interglacial period (i.e., from −9000 ad to about 60 000 ad). LDFs for the global warming climate case represent the maximum during this period.

In deriving LDF values for the different periods of the reference glacial cycle we introduced several assumptions about the future evolution of the system, some of which were realistic and some other pessimistic. The model itself also includes a combination of realistic and cautious assumptions. LDF values obtained from deterministic simulations under these baseline assumptions are here called the baseline LDF values, which were used in the dose calculations for comparison with regulatory dose criteria.

### Methods for Uncertainty and Sensitivity Analyses

Very briefly, we have studied system, model, and parameter uncertainties as well as numerical model integration uncertainties pertaining to the LDF values that we have derived. Furthermore, we carried out sensitivity analyses for time-independent parameters using the results from probabilistic simulations. A detailed description of the methods used is given in the Appendix (Electronic Supplementary Material).

## Results and Discussion

### The Radionuclide Model of the Biosphere

**Fig. 2:**
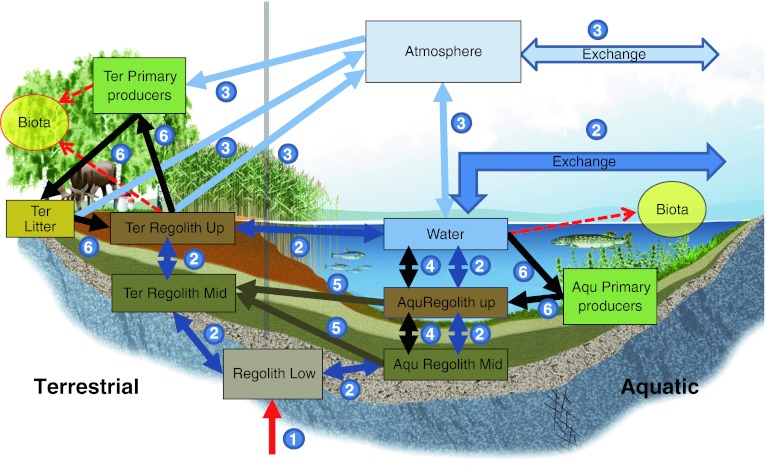
Conceptual illustration of the Radionuclide Model of the biosphere. *Boxes* represent compartments, *thick arrows* fluxes, and *dotted arrows* concentration computations for biota (these are not included in the mass balance calculations). The model represents one biosphere object which contains an aquatic part (*right*) and a terrestrial part (*left*) with a common lower regolith and atmosphere. The release from the geosphere is represented by a *red arrow* (*1*). The radionuclide transport is mediated by different major transport processes, indicated with *dark blue arrows* for water fluxes (*2*), *light blue* for gas fluxes (*3*), *black* for sedimentation/resuspension fluxes (*4*), *dark brown* for wetland growth (*5*), and *green* for biological uptake/decomposition (*6*). Import from and export to surrounding objects in the landscape is represented by *arrows* marked ‘exchange’. The compartments are described in Table 1

The Radionuclide Model of the biosphere is a classical compartment model, where system components are considered internally homogeneous and are represented by distinct compartments. Figure 2 shows a graphical representation of the conceptual model for one biosphere object, in which each box corresponds to a model compartment (see Table 1). The arrows in Fig. 2 represent radionuclide fluxes between compartments and fluxes in and out of the biosphere object, which will commonly have a central depression (in this case a lake). Radionuclide fluxes are linked to the main fluxes of matter in the biosphere, i.e., water fluxes (2 in Fig. 2), gas fluxes (3 in Fig. 2), and particle fluxes (4 in Fig. 2). Radionuclide transfers mediated by biota (6 in Fig. 2), like uptake by primary producers, have also been considered. The arrow (1 in Fig. 2) reaching the lower regolith compartment represents radionuclide releases from the geosphere into the biosphere object. These releases are directed to the deeper parts of the regolith, which at the site normally consists of glacial till directly overlying bedrock.

**Table 1:** Compartments included in the Radionuclide Model of the biosphere

Name	Description
Regolith Low	The lower part of the regolith overlying the bedrock, primarily composed of glacial till
Aqu Regolith Mid	The middle part of the regolith in the aquatic part of biosphere objects, usually consisting of glacial and postglacial clay and gyttja
Aqu Regolith Up	The part of the aquatic regolith with highest biological activity, comprising ca. 5–10 cm of the upper aquatic sediments where resuspension and bioturbation can maintain an oxidizing environment
Ter Regolith Mid	The middle part of the terrestrial regolith, containing glacial and postglacial fine material, i.e., sediments formed in a former seabed/lake bottom environment
Ter Regolith Up	The upper part of the terrestrial regolith which has the highest biological activity, like the peat in a wetland, or the ploughing depth of soil in cultivated land
Litter	Dead plant material overlying the regolith
Water	The surface water (stream, lake, or sea water)
Aqu Primary Producers	The biotic community in aquatic habitats, comprising both primary producers and consumers
Ter Primary Producers	Terrestrial primary producers
Atmosphere	The lower part of the atmosphere where released radionuclides are fully mixed

Radionuclides released to the lower regolith compartment are distributed to the upper layers of the ecosystems by advection and diffusion. Our model of the waterborne transport of radionuclides between compartments is based on detailed hydrological modeling studies with MIKE-SHE (Graham and Butts 2005; Butts and Graham 2008; Berglund et al. 2013b). These studies have shown that the vertical hydrological fluxes in the deep regolith layer of sea basins and bays are minute. Discharge areas at shorelines may, on the other hand, have substantial vertical fluxes with preferential flow paths through areas of higher permeability within a biosphere object, as in wetlands surrounding lakes and streams.

We also take into account the effect of sorption on the advective and diffusive transport of radionuclides. This is done by assuming equilibrium between the pore water and the solid phase of the compartments and using *K*_d_ values to quantify the partition of radionuclides between these phases. Our model also considers the transport of radionuclides absorbed to suspended particles driven by surface water fluxes, sedimentation, and resuspension processes.

The model describes the radionuclide transport mediated by biota by assuming that these fluxes are driven by primary production, in both terrestrial and aquatic ecosystems. Further, we assume that equilibrium is established between the concentration of radionuclides in newly produced biomass and the corresponding environmental media (upper regolith for terrestrial primary producers and water for aquatic primary producers) and use CR values to estimate radionuclide concentrations in this newly produced biomass. This is an improvement over traditional equilibrium plant uptake models, as we represent plant uptake dynamically as a function of growth, while at the same time ensuring that mass balance is maintained (Avila 2006a, b; Andersson 2010; Erichsen et al. 2013).

The model supports simultaneous simulation of aquatic and terrestrial ecosystems and this feature has allowed representation of the continuous development in time of biosphere objects and the whole potentially affected landscape. When applied to each biosphere object the Radionuclide Model has two parts, one aquatic (right side in Fig. 2) and one terrestrial (left side in Fig. 2). We handle the temporal development of an object by varying the sizes and properties of these two parts in accordance with the simulated development of the specific biosphere object, resulting from natural processes such as shoreline displacement, sedimentation, and lake infilling (Lindborg et al. 2013).

The model representation of a biosphere object changes as follows: During the sea stage, when the biosphere object is submerged, there are no terrestrial compartments and all fluxes from the lower regolith are directed to aquatic sediments. During a transitional stage (~500 years), the sea bay is isolated and transforms into a lake, and a wetland starts to develop. Our denomination of the compartments and processes changes as a consequence of a changing environment. For example, the flux of radionuclides from the deep regolith will gradually shift from aquatic sediments to sediments under the wetland. During this phase, saltwater flooding will still occur, although at reducing frequency, and consequently we vary the values of the aquatic model parameters continuously, going from sea to lake values. The surrounding wetland gradually expands into the lake, and the lake sediments are gradually covered by a layer of peat. We represent this process by introducing in the model a flux of radionuclides from the aquatic sediments to the terrestrial regolith (arrows 5 in Fig. 2). The natural end state of the biosphere objects is a wetland, usually drained by a small stream. We assume that this wetland might be converted into agricultural land by future humans at any desired time.

### Model Parameter Values

The Radionuclide Model of the biosphere uses approximately 140 input parameters, of which one-third represent radionuclide- or element-specific properties. The BE values and PDFs used in the safety assessment can be found in Nordén et al. (2010) and Avila et al. (2010). It should be noted that in the Radionuclide Model of the biosphere, compartments are assumed to be internally homogeneous, and a temporal resolution of years was considered to be sufficient for assessing average life-time doses from long-term releases. Thus, parameters were selected to give a yearly mean representing a compartment on the scale of a sea or lake basin.

### Results of Model Simulations for Derivation of LDF Values

**Fig. 3:**
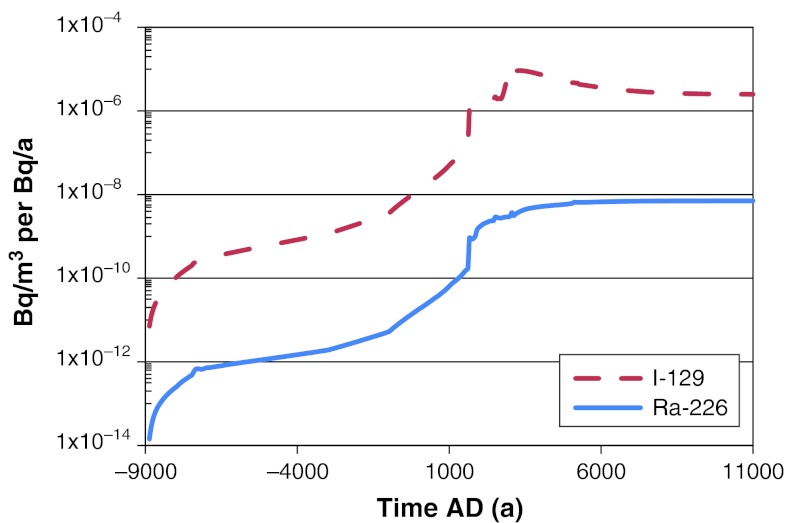
Activity concentrations of ^226^Ra and ^129^I in surface waters. Maximum values across all biosphere objects are shown. The values were obtained from deterministic simulations with a constant release rate of 1 Bq a^−1^ during the interglacial period

**Fig. 4:**
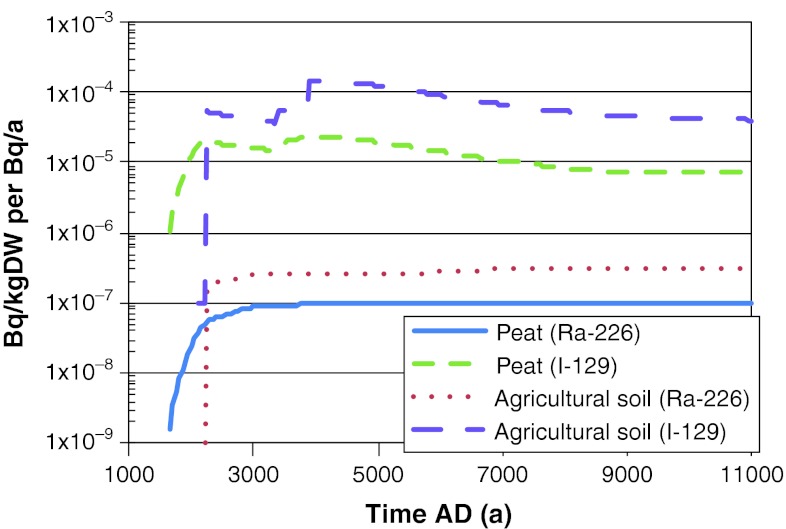
Activity concentrations of ^226^Ra and ^129^I in the upper layer of the mire and in agricultural soil. Maximum values across all biosphere objects are shown. The values were obtained from deterministic simulations with a constant release rate of 1 Bq a^−1^ during the interglacial period

**Fig. 5:**
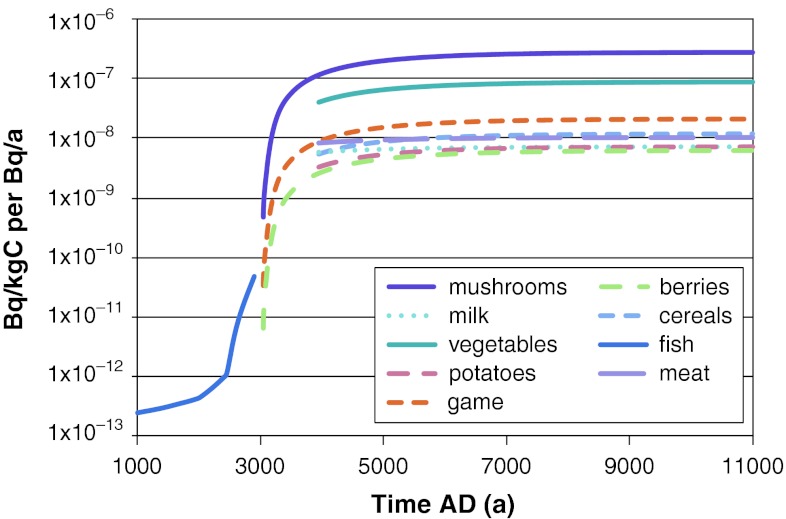
Activity concentrations of ^226^Ra in different food sources in biosphere object 121_03 during an interglacial period. This specific object does not have a lake stage, but it transforms directly into a wetland and therefore concentrations in aquatic products are not shown for this stage

From simulations with the Radionuclide Model of the biosphere we obtained time series of activity concentrations per unit release rate (Bq kg^−1^ dry weight [dw] per Bq a^−1^ or Bq m^−3^ per Bq a^−1^) of each radionuclide in different environmental media (water, sediments, air, and soil). We then used these activity concentrations to calculate activity concentrations in different types of food consumed by humans (Bq kg^−1^ of C per Bq a^−1^). In Figs. 3, 4, and 5 we show examples of time series of ^226^Ra and ^129^I activity concentrations in the environmental media and in human foods. In general, we observed large differences between concentrations per unit release rate obtained for different radionuclides. For example, we observed a difference of two orders of magnitude between the ^129^I and ^226^Ra concentrations per Bq a^−1^ release in surface water (Fig. 3). This difference can be explained by differences in steady-state output of these radionuclides from the lower regolith compartment, which are enhanced by radioactive decay losses of ^226^Ra (due to its shorter half life) during passage through deeper terrestrial compartments; i.e., before reaching the surface water compartment.

**Fig. 6:**
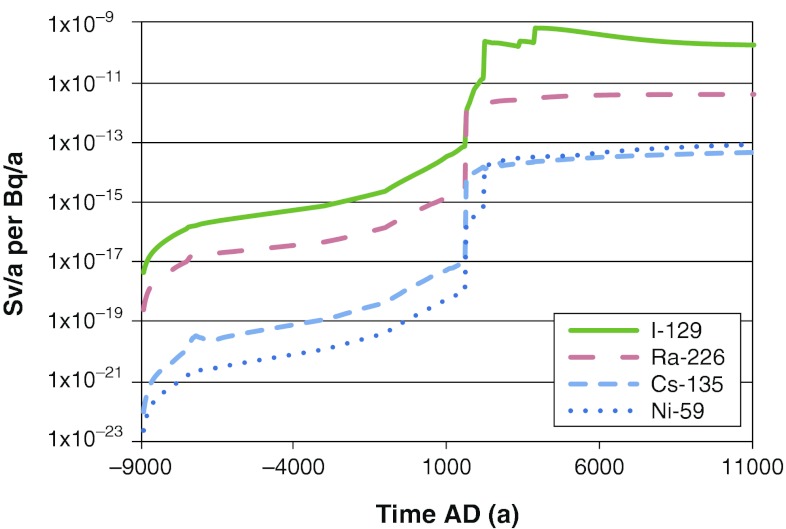
Time series of LDF values for a selection of radionuclides. Maximum values across all biosphere objects are shown. The release rate is 1 Bq a^−1^

From the time series of activity concentrations in the environment, we obtained time series of annual doses per unit release rate (Fig. 6) and from these we could calculate LDF values that were used in the safety assessments.

### Results of Uncertainty and Sensitivity Analyses

Avila et al. (2010) provide detailed results of all uncertainty analyses that we carried out for several radionuclides. ^226^Ra was the radionuclide with the highest contribution to the doses in the SR-Site safety assessment (Kautsky et al. 2013), and some of the results for this radionuclide are presented in the Appendix (Electronic Supplementary Material). As a general conclusion, the use of baseline LDFs would lead to moderately cautious (pessimistic) or realistic estimates (i.e., underestimation of doses would be unlikely).

Nevertheless, if the final risk estimates obtained from dose calculation using baseline LDF values are close to the regulatory limits (as compared with the difference between the baseline LDF values and the expected values from probabilistic simulations), it would be reasonable to make further efforts to reduce the parameter uncertainty of dose-contributing radionuclides.

As explained in the Appendix (Electronic Supplementary Material), we expect that uncertainty in the LDFs could be significantly reduced if the uncertainties in parameters that describe retention in regolith layers (*K*_d_) and uptake by biota (CR) could be reduced to reflect natural variation on the site.

## Conclusions

The model presented here can be applied to simulate the long-term transport and accumulation in the landscape of radionuclides released via groundwater discharges. This model can handle scenarios with spatially distributed releases to a landscape that experiences long-term dynamical changes driven by natural processes such as land rise, climate change, and ecosystems succession. We have successfully applied this model in the SR-Site safety assessment in order to assess doses in scenarios involving both continuous and pulse releases of a large number of radionuclides that might occur in the far future from potential leakages from a geological repository. We have performed a detailed systematic analysis of the uncertainties of long-term simulations with this model, including uncertainties in the future evolution of the biosphere, as well as model and parameter uncertainties.

These analyses have shown that using this model, it was possible to obtain sufficiently cautious dose estimates, which are suitable for use in demonstration of compliance with the risk criterion. However, from the results of the Monte Carlo simulations, it is evident that uncertainties in several element-specific parameters make a significant contribution to the uncertainty in the dose estimates. Hence, there is a potential for reducing uncertainties, in particular with respect to processes describing the partitioning of radionuclides between the solid and liquid phases (as expressed in *K*_d_ values) and biological uptake (as expressed in CR values). Thus, if the final risk estimates obtained with this model are close to the regulatory risk criterion, it would be reasonable to undertake work to further reduce the uncertainty associated with these processes and parameters.

## Electronic supplementary material

Below is the link to the electronic supplementary material.

Supplementary material 1 (PDF 200 kb)
