# Supplementary material for: Model of the Long-Term Transport and Accumulation of Radionuclides in Future Landscapes
Source: Ambio. 2013 Apr 26;42(4):497–505. doi: 10.1007/s13280-013-0402-x (PMC3636369; doi:10.1007/s13280-013-0402-x)
Supplement: Supplementary file 1 — Supplementary material 1 (PDF 200 kb) [file 13280_2013_402_MOESM1_ESM.pdf]

Electronic Supplementary Material

Model of the Long-Term Transport and Accumulation of Radionuclides in Future Landscapes

Rodolfo Avila Moreno, Ulrik Kautsky, Per-Anders Ekström, Per-Gustav Åstrand, Peter Saetre

UNCERTAINTY AND SENSITIVITY ANALYSES

For this study, we have classified uncertainties in the results of model simulations for deriving LDF values into three types: i) System Uncertainties, arising from our inability to make accurate predictions of the long-term development of the biosphere and the future use of that biosphere by humans; ii) Model Uncertainties, arising from our incomplete knowledge of the processes affecting the behavior of radionuclides in the biosphere, which leads to imperfect conceptual models and simplified mathematical representation of the conceptual models; and iii) Parameter Uncertainties, arising from the natural variability of the parameters and from imperfect and insufficient measured data.

In addition, we studied uncertainties associated with errors arising during the numerical integration of the models (Avila et al. 2010). The approach to uncertainty analyses that we adopted is generally consistent with frameworks for analysis of uncertainties applied in disposal programs worldwide (Galson and Khursheed 2007).

System uncertainties have been treated by introducing several assumptions in the derivation of baseline LDF values, some of which are considered cautious and other realistic. The quantification of the effects of system uncertainties has consisted of deriving LDF values under alternative assumptions (Table S1) and comparing these with the baseline LDF values.

Model (conceptual) uncertainty is defined here as the collective uncertainty present in the Radionuclide Model of the biosphere, excluding parameter uncertainty and numerical uncertainty in the integration of the models. Sources of model uncertainty are those assumptions, approximations, or choices that we made in the model used for derivation of baseline LDF values.

The evaluation of model uncertainty consisted of performing simulations with alternative

Table S1 Summary of system uncertainties that have been analyzed. The assumptions made for derivation of baseline LDF and alternative assumptions for the uncertainty analysis are indicated. Adapted from Avila et al. (2010)

Source of uncertainty	Assumptions for baseline LDF	Alternative assumptions
	Development of the biosphere	
**Timing of releases** **(**State of the biosphere in relation to the timing and duration of the releases)	Baseline LDFs are derived from peak dose values from constant unit releases over the whole interglacial period (cautious assumption)	Releases start at different time points and have lower duration
**Localization of releases** (State of the biosphere in relation to the localization of potential releases in the landscape)	Baseline LDFs are maximum across all biosphere objects selected (cautious assumption)	LDF values for each biosphere object and average value across all biosphere objects compared with baseline LDFs
**Climate change** (Effect of different climatic conditions on the LDFs)	Baseline LDF are maximum during the interglacial period and are used for dose calculations during the whole simulation period (cautious assumption)	Simulations for alternative climate conditions, see Avila et al. (2010)
	Human utilization of natural resources	
**Irrigation with well water** (Use of surface and well water for short-term irrigation)	It is assumed that only contaminated surface water is used for irrigation (realistic assumption)	Considering short-term irrigation with well water
**Long-term irrigation** (Use of surface water for long-term irrigation)	Long-term irrigation with surface water is not considered, since it is bounded by accumulation in mire and short-term irrigation. Use of well water for long term irrigation is considered unlikely (realistic assumption)	Considering long-term irrigation with contaminated surface water

Table S2 Summary of model (conceptual) uncertainties that have been analyzed. The assumptions made for derivation of baseline LDF and alternative assumptions for the uncertainty analysis are indicated. Adapted from Avila et al. (2010)

Source of uncertainty	Assumptions for baseline LDF	Alternative assumptions
	Model discretization	
**Size of the biosphere objects**	The smallest identified basin (object 121) was divided into three smaller biosphere objects (cautious assumption)	Simulations with undivided smallest basin, i.e., object 121 as one single biosphere object
**Regolith discretization** (discretization of the lower regolith compartment)	The lower regolith is represented by a single compartment (cautious assumption)	Simulations with a finer discretization of the lower regolith (several compartments)
	Vertical transport and retention of radionuclides in the regolith	
**Diffusion** (Representation of diffusion)	Vertical transport by diffusion considered in a simplified way (simplifying assumption)	Simulations disregarding diffusion
**Advection** (Representation of advective transport from the lower regolith)	In the simulations advective transport increases from sea to lake/terrestrial stages (simplifying assumption)	Simulations assuming constant high advection and constant low advection

assumptions and models (Table S2) to derive alternative LDF values which we then compared with the baseline LDF values.

For parameters the values of which are kept constant during the whole simulation period (time-independent parameters), we studied the effect of their uncertainty on the LDF values by performing probabilistic simulations using Monte Carlo methods. However, we could not use this method to study the effect on the LDF values of the uncertainty in time-dependent parameters, since these variables are strongly correlated with each other. Instead, we evaluated the uncertainty by performing a series of alternative deterministic simulations where we co-varied these parameters as a group. We then compared the LDF values obtained from these simulations with the baseline LDF values.

Methods for sensitivity analysis

For time-independent parameters, we carried out sensitivity analyses using the results from probabilistic simulations. The sensitivity analyses consisted of computing the First Order Sensitivity Index (FOSI) and the Standardized Regression Coefficients (SRC) using the samples generated from the Monte Carlo simulations.

The SCRs are a measure of the importance of the different parameters for a given output, which is obtained from fitting the model predictions for the output to a first-order polynomial dependency on the studied input parameters (Saltelli et al. 2000). The higher the SRC for a parameter, the higher is its effect on the output. A positive SRC value indicates that the input and the output move in the same direction, whereas a negative SRC indicates that they move in opposite directions.

The FOSI is a measure of the contribution of input parameters to the variance of the outputs, obtained by methods based on variance decomposition (Saltelli et al. 2000). The FOSIs consider the only first-order contributions to the variance of the output, i.e., contributions from interactions with other parameters are not taken into account.

Results of uncertainty analyses

Avila et al. (2010) provide detailed results of all uncertainty analyses that we carried out for several radionuclides. These analyses showed that the relative significance of the different uncertainties varied between radionuclides. Here we only present some of the results for ^{226}Ra (Fig. S1), which was the

radionuclide with the highest contribution to the doses in the SR-Site safety assessment (Kautsky et al. 2013).

Figure S1 shows the derived baseline LDF values (central horizontal line) and LDF values derived from supporting simulations that we carried out for evaluating specific sources of system (Table S1) and model (Table S2) uncertainties.

If the LDF obtained for a supporting simulation is below the central line (baseline LDF value), then it can be concluded that, for this specific source of uncertainty, the assumptions made in the derivation of baseline LDF have led to cautious estimates. Values above the line indicate that the use of baseline LDF values might lead to underestimation of doses under specific conditions (depending on the corresponding source of uncertainty). From examination of these plots for all studied radionuclides (Avila et al. 2010), we could make the following observations:

The LDF values derived from simulations to evaluate system and model uncertainties were within the 5th and 95th, and in many cases between the 25th and 75th, percentiles of the LDF obtained from probabilistic simulations. Note that in the probabilistic simulations we used the same system and model assumptions as in the deterministic simulations for derivation of baseline LDF values. This indicates that the overall uncertainty of the baseline LDFs is dominated by parameter uncertainties.

In general the evaluations of system and model uncertainties indicate that the use of the baseline LDFs would lead to cautious (pessimistic) or realistic estimates. This was the case for all studied radionuclides, with one exception: For ^{135}Cs the results indicate that LDFs for a global warming scenario might be higher than the baseline LDF and the 95th percentile from the probabilistic simulations. However, in the safety assessment this has been handled by calculating a separate LDF for the global warming climate alternative. Another exception is the uncertainty in the LDF values for ^{226}Ra associated with the use of well water for irrigation.

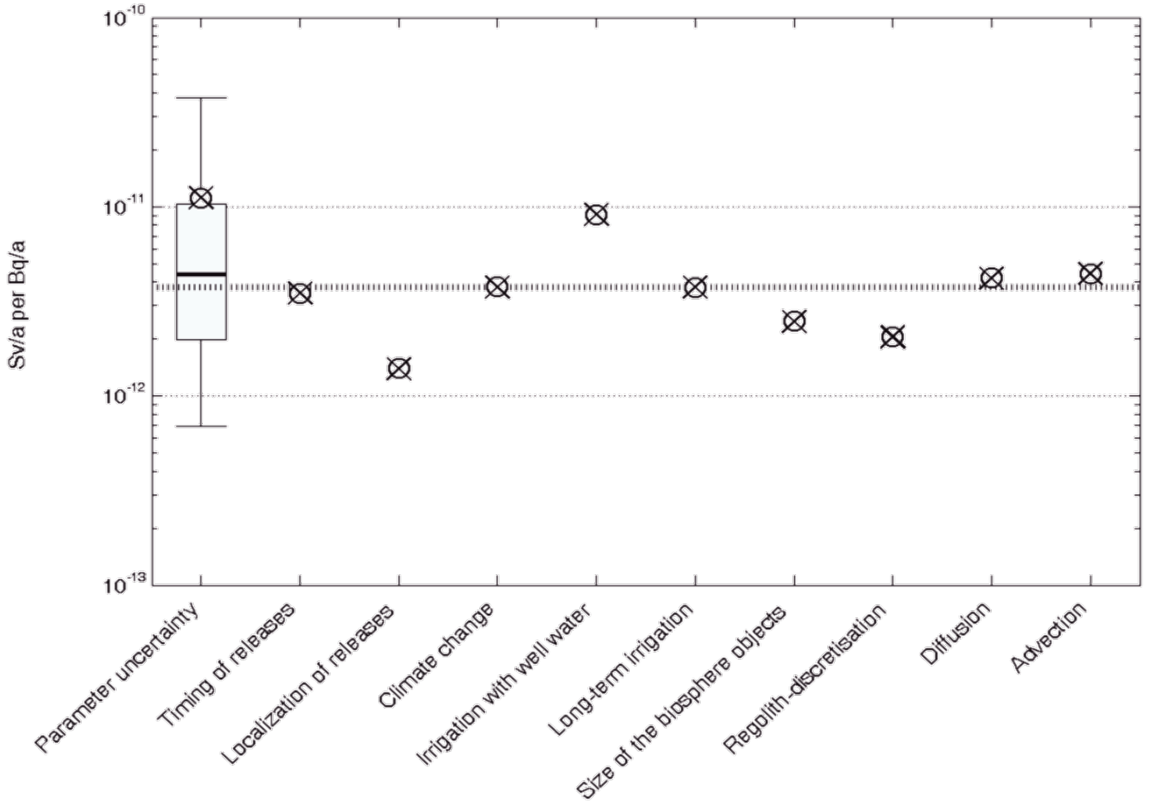

Fig. S1 Results from uncertainty analyses of the maximum LDF values for ^{226}Ra. The central horizontal line corresponds to the baseline LDF and the circles show LDF values obtained from different supporting simulations for evaluation of system (see Table S1) and model (see Table S2) uncertainties. The whisker plot shows the 5th, 25th, median, 75th, 95th, and mean value (circle) of the LDFs obtained from the probabilistic simulations to study parameter uncertainties

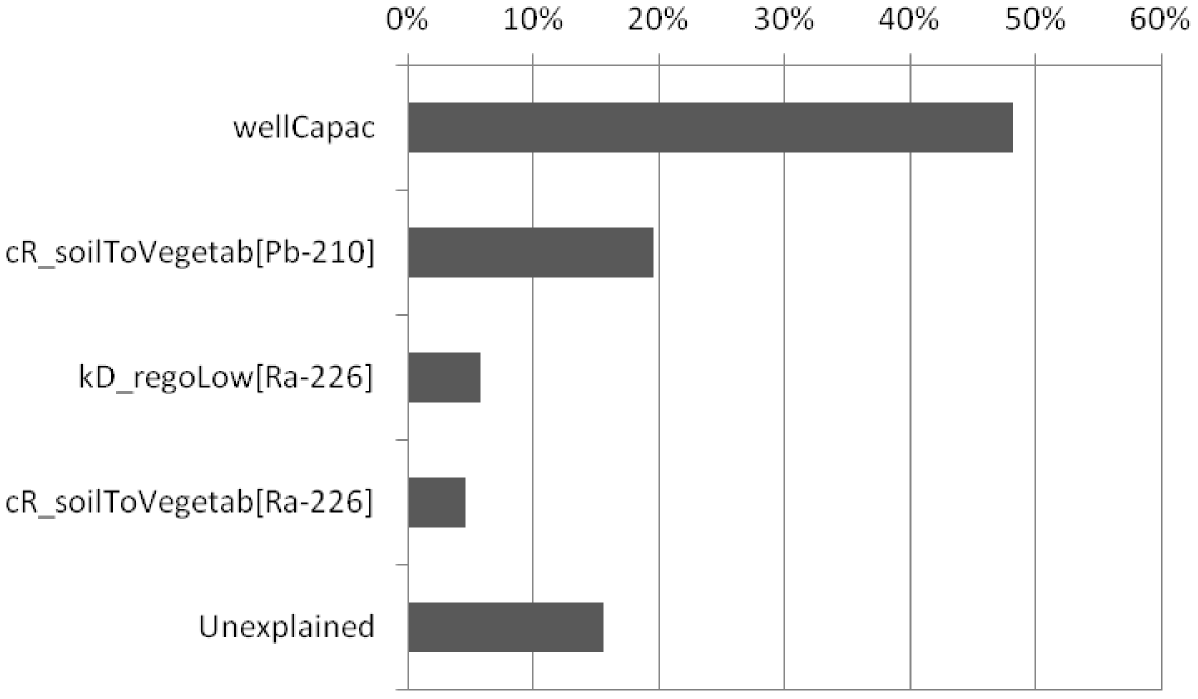

Fig. S2 First-order sensitivity indices of the maximum LDF for ^{226}Ra obtained from probabilistic simulations. The parameters with the highest contribution to the uncertainty of the LDF values are: the capacity of the drilled water well (wellCapac), the concentration ratio from soil to vegetables of ^{210}Po (cR_soilToVegetab[Po-210]), the distribution coefficient of ^{226}Ra in the lower regolith layer (kD_regoLow[Ra-226]), and the concentration ratio from soil to vegetables of ^{226}Ra (cR_soilToVegetab[Ra-226]). The bar titled 'unexplained' represents the fraction of the variance that cannot be explained by first order effects

The degree of cautiousness in the approaches for treatment of system and model uncertainties was moderate, as evidenced by small differences between LDF values from supporting simulations and baseline LDF values. The sources of uncertainties that have been treated most cautiously are those related to the timing and localization of the releases and the discretization of the regolith. The degree of cautiousness varied between radionuclides, but since all LDF values were within the interval from the probabilistic simulations, it can be concluded that the treatment of these uncertainties was not over-pessimistic.

Uncertainties in time-dependent parameters, for example parameters that represent the landscape development, had a limited effect on the uncertainty of the LDF estimates. At the same time, it is evident that uncertainties in time-independent parameters made a significant contribution to the uncertainty in LDF values.

Moreover, the expected values from the probabilistic simulations were systematically higher than the baseline LDF values. However, though a great effort was put into the process to derive meaningful PDFs, information from the site was occasionally insufficient, resulting in PDFs reflecting a wide span reported in the literature rather than the variation expected for the site taking into account expected spatial and time variability within the time frame of the assessments.

Furthermore, the Monte-Carlo sampling did not incorporate correlations between parameters (e.g., the expected negative correlation between Concentration Ratios, CR, for plants and Distribution Coefficients, K_d, for soils). Nevertheless, the difference between baseline LDF values and expected values from the probabilistic simulations gives a good indication of the potential impact of parameter uncertainties.

Thus, if the final risk estimates obtained from dose calculation using baseline LDF values are close to the regulatory limits, (as compared with the difference between the baseline LDF values and the expected values from the probabilistic simulations), it would be reasonable to make further efforts to reduce

the parameter uncertainty of dose-contributing radionuclides.

Results of sensitivity analyses

Although there are differences between radionuclides, uncertainty in parameters that describe retention in regolith layers (K_d) and uptake by biota (CR) explained a large fraction of LDF uncertainty for all radionuclides (see Fig. S2 for ^{226}Ra). An increase in CR values and in K_d values for the upper- and mid-regolith layers was always associated with an increase in LDF, whereas an increase in K_d for the lowest regolith layer was associated with a decrease in LDF.

The primary reason for the large impact of uncertainty in K_d and CR values on the LDF uncertainty was that the distributions of these parameters were typically very wide. We based our estimates of PDFs for these parameters on a combination of site and literature data, which covered a broad range of environments. In this study, we did not take into account possible systematic variations due to, for example, climate, and geographical location, type of ecosystem, or measurement technique.

Hence, the derived PDFs for these parameters are likely to overestimate the natural variation to be expected at the site (Tröjbom and Nordén 2010). We therefore expect that uncertainty in the LDFs could be significantly reduced if the uncertainties in these parameters could be reduced to reflect natural variation on the site. It may also be possible to reduce uncertainties for some radionuclides by describing plant uptake by alternative modeling approaches that are less sensitive to parameter uncertainties.

For ^{226}Ra, the parameter well capacity also showed a large impact on the uncertainty of the dose estimates, mainly because the higher predicted radionuclide concentrations in well water, as compare to surface water. However, reducing the uncertainty in this parameter is relatively easy and straightforward.

REFERENCES

Avila, R., P.-A. Ekström, and P.-G. Åstrand. 2010. Landscape dose conversion factors used in the safety assessment SR-Site. Svensk Kärnbränslehantering AB, SKB TR-10-06, Stockholm, Sweden, Report, 169 pp.

Galson, D. A., and A. Khursheed. 2007. The treatment of uncertainty in performance assessment and safety case development: state-of-the art overview. PAMINA Milestone Report M2.1.1, European Commission. 205 pp. Retrieved January 6, 2013, from http://www.ip-pamina.eu/downloads/pamina.m1.2.1.pdf

Kautsky, U., T. Lindborg, and J. Valentin. 2013. Humans and ecosystems over the coming millennia - overview of a biosphere assessment of radioactive waste disposal in Sweden. *AMBIO*. doi: 1007/s13280-013-0405-7

Saltelli, A., K. Chan, and M. Scott. 2000. *Sensitivity analysis*. Chichester, UK: John Wiley & Sons, 504 pp.

Tröjbom, M., and S. Nordén. 2010. Chemistry data from surface ecosystems in Forsmark and Laxemar-Simpevarp. Site-specific data used for estimation of CR and K_d values in SR-Site. Svensk Kärnbränslehantering AB, SKB R-10-28, Stockholm, Sweden, Report, 145 pp.
